# Structural diversity of supercoiled DNA

**DOI:** 10.1038/ncomms9440

**Published:** 2015-10-12

**Authors:** Rossitza N. Irobalieva, Jonathan M. Fogg, Daniel J. Catanese, Thana Sutthibutpong, Muyuan Chen, Anna K. Barker, Steven J. Ludtke, Sarah A. Harris, Michael F. Schmid, Wah Chiu, Lynn Zechiedrich

**Affiliations:** 1 Graduate Program in Structural and Computational Biology and Molecular Biophysics, Baylor College of Medicine, Houston, Texas 77030 USA; 2 Verna and Marrs McLean Department of Biochemistry and Molecular Biology, Baylor College of Medicine, Houston, Texas 77030 USA; 3 Department of Molecular Virology and Microbiology, Baylor College of Medicine, Houston, Texas 77030 USA; 4 Department of Pharmacology, Baylor College of Medicine, Houston, Texas 77030 USA; 5 School of Physics and Astronomy, University of Leeds, Leeds LS2 9JT, UK

## Abstract

By regulating access to the genetic code, DNA supercoiling strongly affects DNA metabolism. Despite its importance, however, much about supercoiled DNA (positively supercoiled DNA, in particular) remains unknown. Here we use electron cryo-tomography together with biochemical analyses to investigate structures of individual purified DNA minicircle topoisomers with defined degrees of supercoiling. Our results reveal that each topoisomer, negative or positive, adopts a unique and surprisingly wide distribution of three-dimensional conformations. Moreover, we uncover striking differences in how the topoisomers handle torsional stress. As negative supercoiling increases, bases are increasingly exposed. Beyond a sharp supercoiling threshold, we also detect exposed bases in positively supercoiled DNA. Molecular dynamics simulations independently confirm the conformational heterogeneity and provide atomistic insight into the flexibility of supercoiled DNA. Our integrated approach reveals the three-dimensional structures of DNA that are essential for its function.

## Abstract (web summary)

DNA supercoiling strongly affects its metabolism. By electron cryo-tomography, biochemical assays and molecular dynamics simulations, here the authors show that supercoiled DNA minicircles adopt unique and wide distributions of three-dimensional conformations, many with disrupted base pairs.

## Graphical abstract

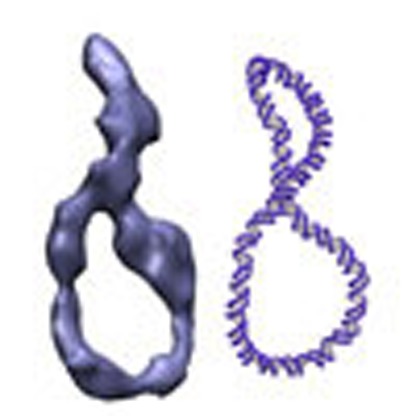

The structure of the B-form DNA double helix has been known for over 60 years^1^, yet DNA metabolism requires deviations, sometimes extreme, from this canonical form^2^,^3^,^4^. Studies of DNA are typically performed on short, linear DNA fragments that cannot be supercoiled; therefore our understanding of DNA is incomplete. In most organisms DNA is maintained in a negatively supercoiled (underwound) state. Positively supercoiled (overwound) DNA is transiently generated during DNA replication and transcription, and, if not promptly relaxed, inhibits these processes^5^. Electron microscopy^6^,^7^,^8^,^9^,^10^,^11^,^12^,^13^ (including electron cryo-microscopy^6^,^8^,^11^,^12^,^13^) and atomic force microscopy^10^,^14^ have previously provided significant insight into the structure of negatively supercoiled DNA. Here we build on those earlier studies by covering a broader range of supercoiling, including positive supercoiling.

Using a combination of approaches we here show that supercoiling causes DNA to adopt large conformational variability. Unpaired bases and localized distortions contribute to this variability both by relieving torsional strain and by providing flexible hinges. Positive and negative supercoiling are accommodated differently. The biological implications of these findings are discussed.

## Results

### 336 bp minicircles accommodate a wide range of supercoiling

**Figure 1:**
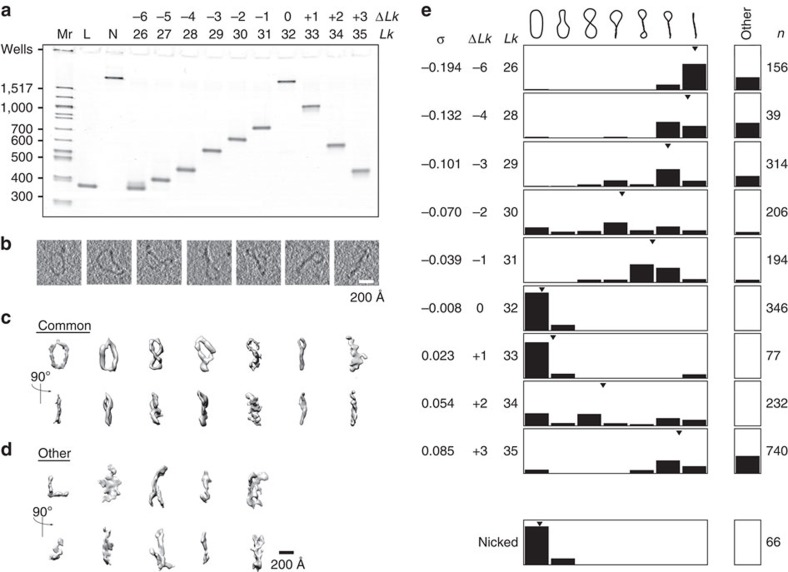
Effect of supercoiling on the structure of minicircle DNA. (**a**) Individual 336 bp minicircle topoisomers were isolated and analysed by polyacrylamide gel electrophoresis in the presence of 10 mM CaCl_2_. Mr: 100 bp DNA ladder, L: minicircle linearized by EcoRV, N: minicircle nicked by Nb.BbvCI. (**b**) Projections of cryo-ET subtomograms of hydrated 336 bp DNA minicircles of the *Lk*=34 topoisomer. (**c**) Commonly observed shapes were open circle, open figure-8, figure-8, racquet, handcuffs, needle, and rod, each of which are shown in orthogonal views. (**d**) Other shapes observed, especially in the more highly supercoiled topoisomers. (**e**) Shape frequency distribution plot for each topoisomer population (n=number of minicircles analysed). A weighted average for each topoisomer, approximating the average degree of compactness, is denoted by the black triangle. The weighted average was calculated by assigning each conformation a value that increased in line with compactness. Open circles were given a value of 1, open figure-8 s a value of 2, figure-8 s as a value of 3, and so on. The relative fraction of each was subsequently used to determine the average degree of compactness. *Lk*, Δ*Lk* and superhelical density (σ) for each topoisomer are shown (see Supplementary Note 1).

Minicircles containing 336 bp were selected for this study because they are representative of the supercoiled DNA loops found in nature^15^,^16^,^17^,^18^. Furthermore, these minicircles are small enough to allow isolation of suitable amounts of individual topoisomers (Fig. 1a), yet large enough to yield an ample spread of ten unique topoisomers (Supplementary Fig. 1). In its relaxed state, the two strands of a 336 bp DNA circle wrap around each other 32 times. This number is known as the linking number (*Lk*). The other topoisomers deviate (Δ*Lk*) from the *Lk* of the relaxed topoisomer (*Lk*=32, Δ*Lk*=0) in steps of 1. We generated and isolated six different negatively supercoiled minicircle topoisomers (*Lk*=31 through 26, Δ*Lk*=−1 through −6), three different positively supercoiled topoisomers (*Lk*=33 through 35, Δ*Lk*=+1 through +3), relaxed (*Lk*=32, Δ*Lk*=0), nicked and linearized minicircles (Fig. 1a, Supplementary Table 1).

All nine of the supercoiled minicircles were subjected to and relaxed by human topoisomerase IIα (htopoIIα) (Supplementary Fig. 2). This relaxation demonstrated the biological activity of the minicircles, confirmed their *Lk* designations, and verified that there was no cross-contamination among topoisomers. Because htopoIIα relaxes in characteristic steps of two *Lk*, all of the minicircles with even *Lk* relaxed to Δ*Lk*=0. The odd-numbered *Lk* topoisomers relaxed to a mixture of Δ*Lk*=−1 and Δ*Lk*=+1.

### Wide variety of minicircle DNA conformations

**Figure 2:**
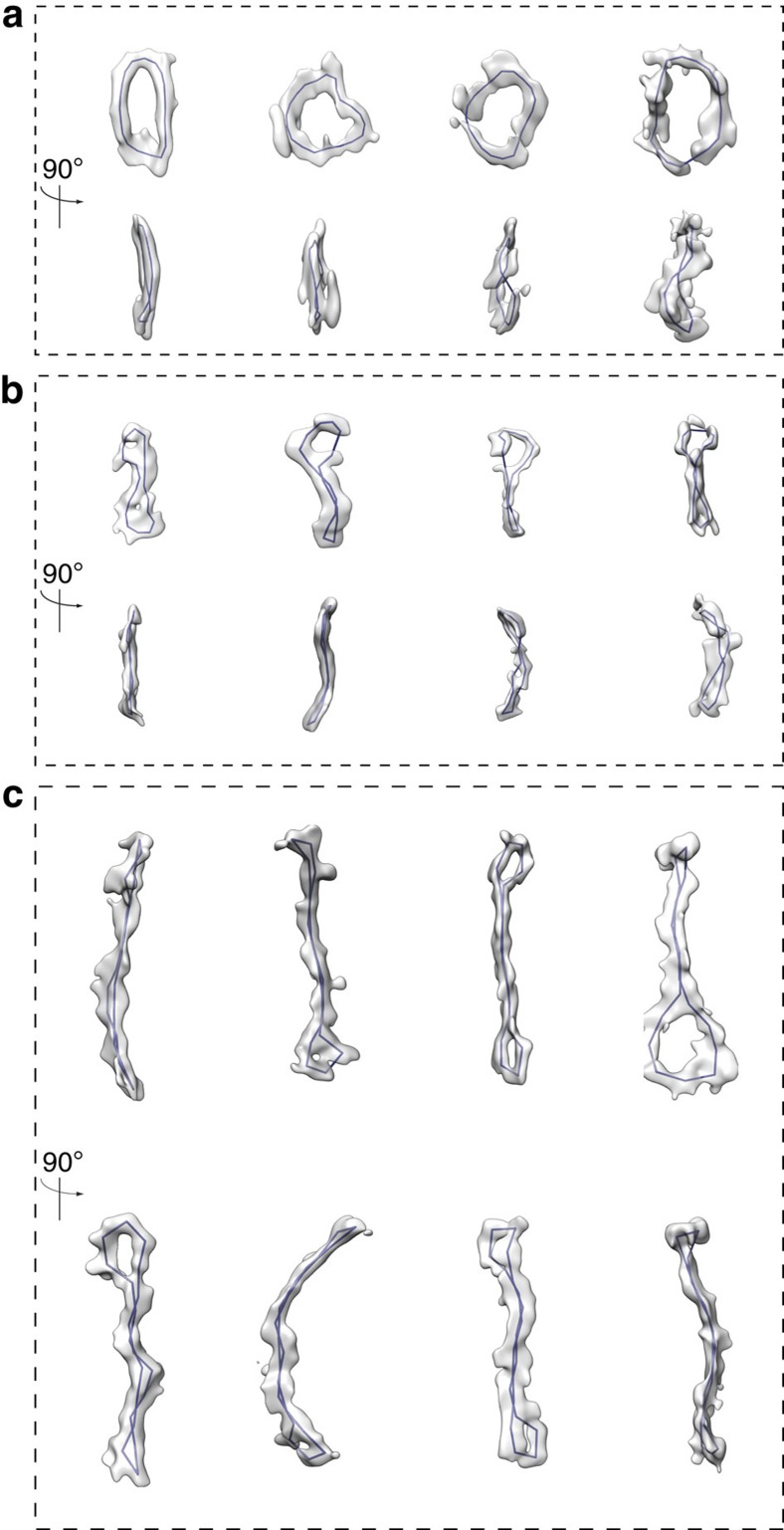
Computational tracing of 336 and 672 bp minicircles. (**a**) Docking of 336 bp traces into the cryo-ET densities of open circles. Traces (purple lines) were generated by docking circular strings of length 336 bp into the density maps. Each trace was then used to isolate the minicircles (grey surfaces) from the cryo-ET density maps. (**b**) Docking of 336 bp traces into the cryo-ET tomograms of writhed minicircles following the same protocol as for the open circles. (**c**) Docking of double-length (672 bp) traces into the cryo-ET tomograms following the same protocol as for 336 bp.

Electron cryo-tomography (cryo-ET) was used to obtain three-dimensional (3D) structures of the different minicircles embedded in vitreous ice. Before freezing, the purified topoisomers were either incubated on ice or at room temperature for at least 15 min in an attempt to allow the DNA to reach conformational equilibrium. Specimen vitrification^19^ occurs at a rate of freezing (10^6^ °C per second) that should be fast enough to preclude temperature-dependent structural alterations^20^. Projection images were obtained with an electron microscope by incrementally tilting the specimen stage. These images were subsequently reconstructed into 3D volumes (tomograms) containing the minicircles (Supplementary Movie 1). Thus, these data represent snapshots of the DNA molecules at the instant of freezing. We computationally generated traces of 336-bp minicircle DNA backbones and fitted these to the observed densities in the tomograms, confirming that each subvolume represented a single 336 bp minicircle (Fig. 2a,b, Methods). In addition, as a control, we visualized double-length minicircles (672 bp) and computationally fitted a 672 bp backbone to the observed densities, confirming their length and thus providing additional validation of the approach (Fig. 2c).

A broad mixture of 3D conformations was observed for each purified 336-bp minicircle topoisomer (Fig. 1b,e). Given the rigidity of DNA at this short length^21^, it is remarkable that the DNA was able to contort into such a wide variety of conformations. The heterogeneity observed indicates that the DNA structure is in dynamic equilibrium, driven by both the torsional stress of supercoiling and by Brownian motion^13^. These results illustrate the power of cryo-ET to visualize individual molecules in solution and capture their conformational variability. Furthermore, cryo-ET data contained sufficient detail to visualize that, as expected, only right-handed crossovers were observed in negatively supercoiled DNA and only left-handed crossovers were observed in positively supercoiled DNA.

Most of the observed minicircle conformations could be classified into the following empirical categories, in order of increasing compaction: open circle, open figure-8, figure-8, racquet, handcuffs, needle, and rod (Fig. 1b,c). Examples of the handcuffs and needle conformations are shown in Supplementary Movie 2 and 3. It is interesting to note that minicircles in topologically distinct topoisomers sometimes adopted the same general conformation (for example, rods found in the Δ*Lk=*−1 and Δ*Lk=*−6 topoisomers appear similar). In these cases, DNA supercoiling must be accommodated in different ways that result in the same general 3D shape. Some minicircles, especially those with larger Δ*Lk* (either negative or positive) adopted alternative shapes (‘other’ in Fig. 1d).

Deviations from relaxed DNA may be manifested as changes in twist, the coiling of the DNA about the helical axis, or writhe, the coiling of the double helices about each other. Changes in twist result in torsional strain whereas changes in writhe result in bending strain. Because we observed multiple DNA conformations in supercoiled DNA—ranging from open to highly writhed—different degrees of twist and writhe must simultaneously exist within the same topoisomer population. DNA bending, and thus writhe, is thought to be more difficult to accommodate for short DNA lengths. Minicircles twice the length (672 bp) with equivalent supercoiling (Δ*Lk*=−4) to the Δ*Lk*=−2, 336 bp minicircle all appeared highly writhed (Fig. 2c). In comparison the Δ*Lk*=−2, 336 bp topoisomer displayed both open and writhed conformations (Fig. 1e). Conversely, smaller minicircles of 94–158 bp have no appreciable writhe^11^,^12^,^13^. Minicircles (336 bp) are, therefore, an ideal size for exploring the relationship between twist and writhe.

As mentioned above, linear DNA is rigid in the aforementioned lengths. Theory therefore predicts that very small circles should be perfectly round. Significant deviations from a perfect circle will require non-uniform distribution of bending along the DNA length. Very small circles already have considerable bending strain. Localized variations in bending should therefore be energetically unfavourable. Ellipticity was measured previously in 94–158 bp DNA minicircles and averaged between 1.1 and 1.5 (refs 11, 12, 13). Increased ellipticity was attributed to the appearance of hyperflexible kinks within the DNA^13^. Thus ellipticity is one measure of conformational variability. To compare our data to those previous studies, we measured ellipticity in a subset of our observed open circles. Significant numbers of minicircles with an open circle shape were found in the following topoisomer populations: Δ*Lk*=−2, 0, +1, +2, +3, and nicked. The measured ellipticity values ranged from 1.1 to 2.6. This larger deviation from circularity is attributable to the longer length of our 336 bp minicircles. We also observed potential differences with supercoiling in the dimensions of the open circles (Supplementary Fig. 3).

**Figure 3:**
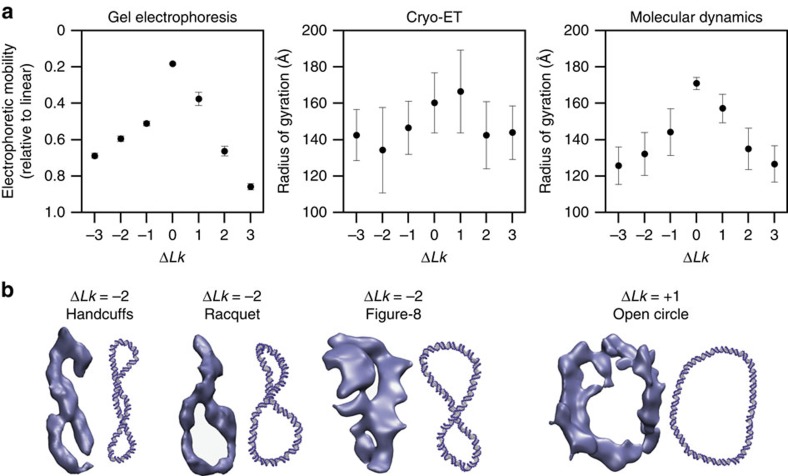
Comparison of electrophoretic mobility and radius of gyration values. (**a**) Left, distance each topoisomer migrated during polyacrylamide gel electrophoresis, measured from the well to the centre of the band (Fig. 1 a), relative to the migration of the linearized 336 bp minicircle. Data shown are the mean values from three separate gels run under identical conditions. Middle, average radius of gyration values obtained from cryo-ET density maps for each topoisomer (*n*=23, 78, 40, 60, 47, 56 and 159 for topoisomers Δ*Lk*=−3, −2, −1, 0, 1, 2 and 3, respectively). Right, radius of gyration (averaged over time) in continuum solvent MD simulations for each topoisomer. Error bars for each of the three graphs represent s.d. values. (**b**) Comparison of cryo-ET data and the equivalent conformations as observed in MD simulations. Examples from negatively supercoiled (Δ*Lk*=−2) and positively supercoiled (Δ*Lk*=+1) topoisomers are shown. MD simulation data are depicted as double-stranded DNA backbone traces.

The conformations adopted by each topoisomer are shown as frequency distribution plots (Fig. 1e). A weighted average of the shape frequency distribution (denoted by arrowheads on each plot) approximates the average degree of compactness. Similarly, electrophoretic mobility provides a measure of relative compaction^22^. By both measures increased negative or positive supercoiling leads to a shift in the distribution towards more compact shapes. To provide a more quantitative measure of compactness, radius of gyration values were measured from the cryo-ET density maps (Fig. 3a, middle). These values were consistent with the electrophoretic mobilities (Fig. 3a, left).

### Molecular dynamics simulations provide atomistic insight

To further understand the conformational fluctuations of the topoisomers, we performed molecular dynamics (MD) simulations (Supplementary Movie 4 and 5; Supplementary Tables 2 and 3). Generalized Born continuum solvent simulations, which, in the absence of viscous damping enable rapid configurational sampling in a relatively short computational time, revealed a wide variety of conformations for each topoisomer. The simulation results suggest that each supercoiled topoisomer undergoes large fluctuations in writhe (Supplementary Movie 4 and Supplementary Fig. 4) and, hence, in the level of compaction. The radius of gyration values, averaged over the MD simulations, showed the same trends with changing superhelical density as the gel electrophoretic mobility and the radius of gyration values extracted from the cryo-ET data (Fig. 3a). Conformations that were observed in the cryo-ET data were also observed in the MD simulations (Fig. 3b), providing insight into how conformations may interchange. The consistency between structural and computational results established confidence for the observation of simultaneous co-existence of multiple conformations of each topoisomer.

Across all levels of supercoiling, differences were observed for negative versus positive Δ*Lk*. Although the Δ*Lk*=−1 and Δ*Lk*=+1 topoisomers might be expected to migrate with similar electrophoretic mobility, Δ*Lk*=−1 migrated much faster on the gel (Fig. 1a). In addition, cryo-ET data revealed that the Δ*Lk*=−1 topoisomer adopted a spread of conformations that included predominantly compact forms (Fig. 1e). The Δ*Lk*=+1 adopted mostly open conformations, similar to the nicked and relaxed minicircles (Fig. 1e). The presence of a small fraction (∼10%) of compact forms may explain why, on average, the Δ*Lk*=+1 topoisomer migrated slightly farther on the gel than the nicked or relaxed minicircles.

Extending the comparison to Δ*Lk*=−2 and Δ*Lk*=+2, we observed broad distributions of conformations, such that every shape category described in Fig. 1c was seen. Overall, the distribution trends for the Δ*Lk*=−2 and Δ*Lk*=+2 were similar. One noteworthy difference, however, was in the relative proportions of the figure-8 and racquet conformations. Racquets were observed ∼3-fold more frequently than figure-8 s for the Δ*Lk*=−2 topoisomer; figure-8 s were ∼5-fold more frequent than racquets for Δ*Lk*=+2. This observation implied structural differences between the two topoisomers, which we verified biochemically as described below.

Further underwinding (Δ*Lk*=−3, −4 and −6) resulted in an additional shift in the distribution toward more compact shapes observed in the cryo-ET data and a concomitant increase in electrophoretic mobility. The increase in electrophoretic mobility between consecutive topoisomers was more pronounced for positive than for negative supercoiling (Fig. 1a). A similar trend was observed for the shift in the conformational distribution between consecutive topoisomers (Fig. 1e). For Δ*Lk*=+3, 39% of the minicircles adopted unusual shapes (‘other,’ Fig. 1d), which were not observed in the Δ*Lk*=+2 population (Fig. 1e), indicating that a sharp structural transition occurs between these two topoisomers.

### Probing for base-pair disruptions and localized denaturation

**Figure 4:**
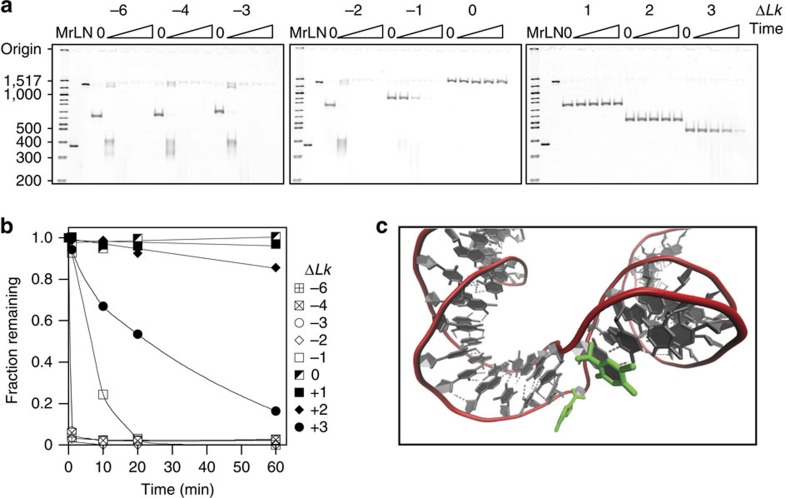
Effect of supercoiling on DNA base accessibility. (**a**) Minicircle DNA incubated with nuclease Bal-31. Over time, samples were removed, quenched by the addition of stop buffer and the products analysed by polyacrylamide gel electrophoresis. Mr: 100 bp DNA ladder, L: linearized 336 bp DNA, N: nicked 336 bp minicircle. (**b**) Graphic representation of the data shown in (**a**) Fitted lines are for visualization purposes only. (**c**) MD simulation of the Δ*Lk*=−3 topoisomer in explicit solvent. Splayed bases were found at a sharp bend of a needle conformation. This may be a potential atomistic explanation for Bal-31 susceptibility of negatively supercoiled topoisomers.

Many of the observed 3D conformations appear to contain sharply bent or kinked DNA. One way that sharp bending may be facilitated is through localized distortions and disruptions of the helix. To probe for and quantify such helix disruptions, we used nuclease Bal-31. Bal-31 has endonuclease function on exposed, unpaired DNA bases (for example, kinks, nicks, gaps, single-stranded regions and B–Z junctions)^23^,^24^. All underwound topoisomers and the most overwound Δ*Lk*=+3 topoisomer had some exposed, unpaired bases, as measured by their Bal-31 sensitivity (Fig. 4a). The negatively supercoiled Δ*Lk*=−2, −3, −4 and −6 topoisomers were linearized within the first minute of Bal-31 incubation and subsequently degraded (Fig. 4b). The Δ*Lk*=−1 topoisomer was cleaved at a much slower rate, requiring 20 min to degrade (Fig. 4b). Consistent with their Bal-31 sensitivity, the electrophoretic mobility of the negatively supercoiled topoisomers shifted when incubated with glyoxal, a small molecule that traps exposed bases (Supplementary Fig. 5). The relaxed (Δ*Lk*=0) and Δ*Lk*=+1 topoisomers resisted Bal-31 digestion, and the Δ*Lk*=+2 topoisomer was a very poor substrate (Fig. 4b). Considering that positive supercoiling should inhibit strand separation, it was surprising that the Δ*Lk*=+3 topoisomer was efficiently cleaved by Bal-31 (Fig. 4a) and this topoisomer was almost completely degraded within an hour (Fig. 4b). Single molecule manipulation studies previously uncovered a structural variation of DNA when it was extremely overwound^25^. The researchers proposed that overwinding may result in an inside-out DNA conformation with the backbones wrapped around each other on the inside and unpaired bases on the outside of the helix. Chemical probing confirmed the presence of unpaired bases. This DNA conformation, named Pauling DNA (P-DNA), was only detected when the DNA was under high tension and writhe was suppressed^25^. The Bal-31 sensitivity of Δ*Lk*=+3 coincides with the dramatic structural changes observed by cryo-ET for this topoisomer (Fig. 1e). Although the presence of exposed bases may imply P-DNA, there are alternative explanations for Bal-31 sensitivity. A probable explanation for the exposed bases is denaturation resulting from sharp bending. Indeed, sharp bending is a feature of the highly writhed conformations of this topoisomer (see below).

To provide an atomistic interpretation of the cryo-ET density for each topoisomer and to explore how localized distortions may alter the structural properties of DNA, we performed explicitly solvated MD simulations (Supplementary Movie 6–9). Because the inclusion of solvent molecules in the calculations slows down configurational sampling, these simulations were performed starting from conformers most commonly observed in the continuum solvent MD. The explicit solvent simulations allowed increased conformational diversity and could be directly fitted to the cryo-ET density maps (Fig. 3b and Supplementary Movie 10–13). Whereas restraints were imposed on the hydrogen bond interactions between complementary base pairs in the simulations in implicit solvent, these restrictions were removed for simulations in explicit solvent. This removal of restraints allowed single-stranded regions to form within the minicircles under sufficiently high levels of torsional or bending stress. Accordingly, in simulations of the Δ*Lk*=−3 topoisomer, a kink emerged at the apex of a highly writhed minicircle, where the hydrogen bonding between complementary base pairs was disrupted (Fig. 4c). Similar base-pair disruptions observed by MD simulations have been reported for bent DNA^26^,^27^ and for underwound linear DNA^28^. These computational results are consistent with the experimental observation that the Δ*Lk*=−3 topoisomer is under a high degree of torsional strain, but additionally predicts that base-pair separation may occur at bent apices. Vologodskii and co-workers found that very small DNA minicircles (64–65 bp) were susceptible to Bal-31 cleavage, which was attributed to kinking resulting from the inherent bending strain^29^. Base-pair disruption has been observed in MD simulations of positively supercoiled DNA, either because of the formation of P-DNA^28^ or because of sharp bending in very small DNA circles^27^.

**Figure 5:**
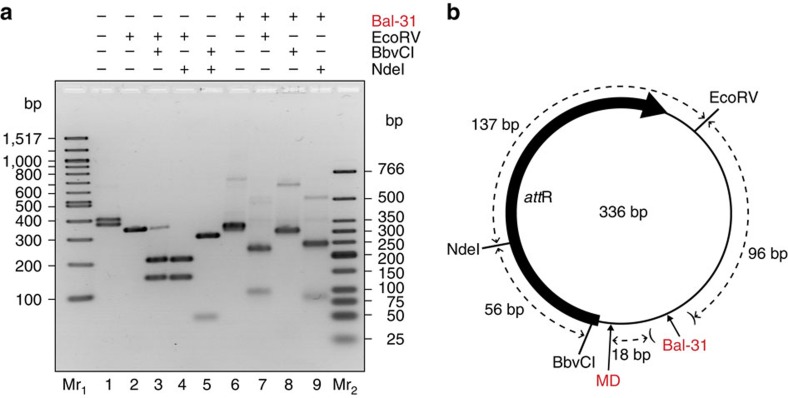
Mapping Bal-31 cleavage. To determine whether Bal-31 cleavage occurs at multiple sites or at a preferred site, the Δ*Lk*=−6 topoisomer was cleaved with Bal-31 and various restriction enzymes. (**a**) Products were separated by agarose gel electrophoresis. Left, (lanes 1–5), control reactions, mc336 (approximately equal mixture of Δ*Lk*=−2 and Δ*Lk*=−3 topoisomers) with combinations of the various restriction enzymes (as indicated) to generate fragments of known DNA lengths. Right, (lanes 6–9), Δ*Lk*=−6 topoisomer cleaved first with Bal-31, followed by a restriction enzyme (as indicated). Mr_1_: 100 bp DNA ladder, Mr_2_: Low molecular weight DNA ladder. (**b**) Map of the minicircle sequence showing the positions of the restriction enzymes used, the estimated location of Bal-31 cleavage (with parentheses indicating the range), and the location of the observed base-pair breaking in MD simulation of the Δ*Lk*=−3 topoisomer.

The propensity for base-pair separation depends on DNA sequence^30^,^31^. We used the WebSIDD algorithm^32^ to predict base-pair separation in our 336 bp minicircle, which identified a short region within *att*R to have a higher probability of duplex destabilization. Because it was very susceptible to Bal-31, the Δ*Lk*=−6 topoisomer was probed. In contrast to the prediction, Bal-31 predominantly cleaved ∼180° away from the anticipated destabilization sequence (Fig. 5). Increased flexibility brought about by localized denaturation at the Bal-31-susceptible site should enable the DNA to be sharply bent. The majority of conformations observed for the Δ*Lk*=−6 topoisomer were sharply bent or kinked (for example, racquets and rods). Kinking at this first site may induce kinking at the site diametrically opposite. Similar cooperative effects between distant sites were seen by Stasiak and co-workers, who observed sequential, cooperative kinking in torsionally strained minicircles at sites located ∼180° apart along the DNA circumference^13^. An additional influence of the location of Bal-31-susceptible sites may be sequence-dependent DNA flexibility and curvature. Sequences with increased flexibility and/or curvature are preferentially localized to superhelical apices^33^ where the DNA is most sharply bent. A short region in *att*R is known to have modest intrinsic curvature^34^,^35^. This curvature may be preferentially located at a superhelical apex^36^, thereby positioning the sequence located ∼180° apart along the DNA circumference at the diametrically opposite superhelical apex. Localized denaturation may be necessary to accommodate the sharp bending at the apex, thereby generating a Bal-31-susceptible site. The kink defect observed by MD at a bent apex (Fig. 4c) is relatively close to the Bal-31 cleavage site (Fig. 5b), consistent with the hypothesis that Bal-31-susceptible sites are localized to the superhelical apices.

## Discussion

Our data collectively demonstrate that supercoiled DNA is able to adopt a wide variety of conformations, the proportions of which depend upon the level of supercoiling. Each topoisomer appears to migrate as a single discrete electrophoretic band (Fig. 1a), suggesting that each individual DNA molecule fluctuates among the different conformations seen by cryo-ET. If there were no interchange among conformations, we would expect multiple discrete bands (representing the different meta-stable conformations), a smear, or a more diffuse band. MD simulations provided further evidence of the dynamic nature of supercoiled DNA and insight into how the conformations may interconvert.

Each topoisomer displayed a wide variety of conformations but the relative proportion of these depended on *Lk*. Because of rapid vitrification, the structures observed should represent a snapshot of the highly dynamic DNA molecule. More highly writhed conformations were observed with increasing negative or positive supercoiling. These trends show how supercoiling drives the dynamic equilibrium and determines how frequently and for how long each minicircle adopts a particular conformation. Somewhat surprisingly, a small number of open circles were observed even in the Δ*Lk*=−6 topoisomer. For these open circles the Δ*Lk* must be partitioned almost entirely into twist.

**Figure 6:**
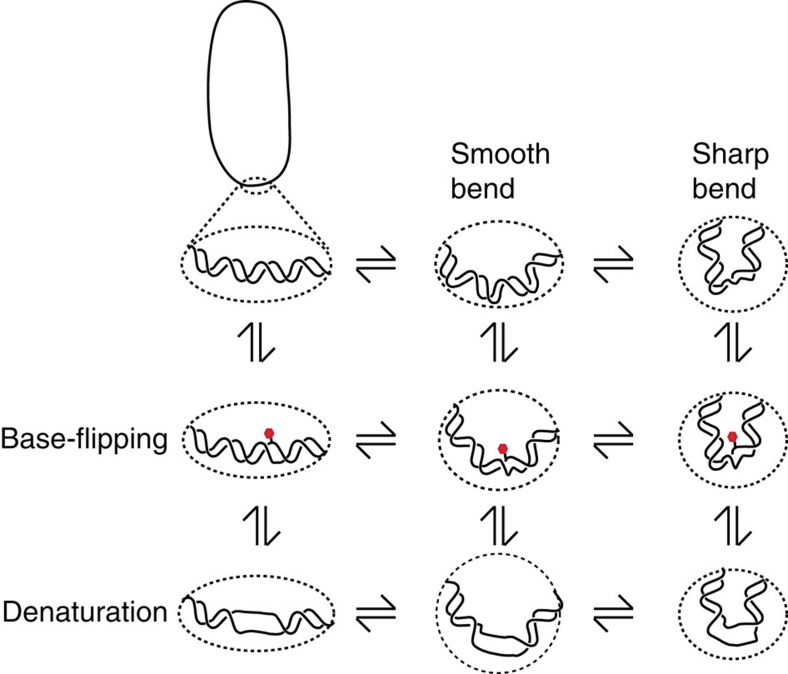
Model for how DNA accommodates supercoiling. Comparison of smooth and sharp bending and the effect of localized denaturation. Images represent a more detailed view of the local structure at the bend. For smooth bending, writhe-mediated bending is regular with bending strain more evenly distributed. Base flipping may generate flexible hinges, allowing DNA to bend more sharply or kink. Alternatively, writhe-mediated sharp bending may lead to disruption of base pairs, even in positively supercoiled DNA. More extensive denaturation may release torsional strain and allow DNA to adopt more open conformations. Denaturation bubbles also provide a flexible joint allowing DNA to kink.

Sensitivity to Bal-31 cleavage revealed the presence of exposed bases when the DNA was negatively supercoiled, and also when highly positively supercoiled. On one hand, localized Bal-31-susceptible distortions create flexible hinges that allow DNA to assume conformations that would be energetically unfavourable if the bases remained paired. On the other hand, extreme writhe and concomitant bending at the apices lead to kinking and localized distortions, even in positively supercoiled DNA. More extensive denaturation relieves torsional strain, one potential explanation for why a small number of open circles are observed in the Δ*Lk*=−6 topoisomer. The release of torsional strain through denaturation may also explain why more open circles are observed in the Δ*Lk*=−2 topoisomer than the Δ*Lk*=−1 topoisomer (Fig. 1e). The interplay between localized denaturation and the sharp bending required to achieve the observed conformations is summarized in Fig. 6. Mapping of the cleavage site of Bal-31 suggested that sharp bending at one location may in turn lead to localized distortions at a site located ∼180° away along the perimeter of the minicircle, similar to the cooperative kinking model^13^.

Our results provide a glimpse into the dynamics and structure of DNA *in vivo* not captured by experiments using short, linear DNA. The situation described in our paper is most comparable to unconstrained DNA, which constitutes a significant fraction of the genomes of *Escherichia coli*, humans, and presumably other organisms^37^,^38^,^39^. Supercoiling, even in the constrained portions of chromosomes, modulates bound architectural proteins. In turn, the bound proteins influence the conformational variability of supercoiling. A protein may be able to exploit the conformational variability of supercoiled DNA by transiently stabilizing one of the conformations, allowing switching between active and inactive states. In actively replicating and transcribing cells, supercoiling fluctuation serves multiple purposes. Positive supercoiling ahead of RNA polymerase facilitates histone dissociation during chromatin remodelling^40^ and important regulatory elements, such as the FUSE element in the c-myc promoter^3^,^41^ or the putative hairpin in the N4 promoter of vRNAP^42^ are tunable by supercoiling.

Six decades after the elucidation of its double helical structure, DNA continues to surprise us by revealing new information. Our cryo-ET, biochemical, and computational studies show the astounding versatility and dynamism of DNA depending on the degree of supercoiling. DNA simultaneously exists in a largely inactive B-form with bases tucked in and protected and an active, highly varied structure with exposed bases. Our data provide relative comparisons of supercoiling-dependent twisted, writhed, curved, and kinked conformations and associated base exposure. Each of these structural features may be differentially recognized by the proteins, nucleic acids, and small molecules that modulate DNA metabolic processes.

## Methods

### Chemicals and reagents

ATP, dithiothreitol (DTT), DNase I, ethidium bromide, glyoxal and RNase A were purchased from Sigma Aldrich (St Louis, MO). Acrylamide, ampicillin, chloroform, isopropyl beta-D-thiogalactoside, sodium chloride and sodium citrate were purchased from Fisher Scientific (Pittsburgh, PA). BbvCI, EcoRV, Nb.BbvCI, NdeI, Nuclease Bal-31, T4 DNA Ligase, low molecular weight DNA ladder and 100 bp DNA ladder were purchased from New England Biolabs (Ipswich, MA). Proteinase K was purchased from Roche Molecular Biochemicals (Mannheim, Germany). All other chemicals were purchased from VWR International (West Chester, PA).

### Expression and purification of HMfB

The expression construct pKS323 (ref. 43) was transformed into CGSC-10851 and cells were grown at 37 °C to an OD_600_=0.4 in Luria–Bertani broth supplemented with 100 μg ml^−1^ ampicillin. HMfB expression was induced by the addition of 0.4 mM isopropyl beta-D-thiogalactoside and expression continued for 16 h. Cells were harvested by centrifugation, resuspended (2 ml per gram of cells) in 50 mM Tris-HCl, pH 8.0, 100 mM NaCl, 2 mM Na_2_HPO_4_ and lysed by two passages through a French pressure cell press at 20,000 psi. Cell debris was removed by centrifugation at 30,000*g* for 30 min. Cleared lysates were transferred to new tubes and centrifuged for another hour at 30,000*g*. The supernatant, after addition of 5 mM MgCl_2_ and 0.1 mM phenylmethylsulfonyl fluoride, was treated with 20 μg ml^−1^ DNase I at 37 °C for ∼3 h. NaCl was added to a final concentration of 3 M and the mixture was heated slowly to 80 °C for 10 min, resulting in precipitation of native *E. coli* proteins. The mixture was cooled to room temperature, filtered through several layers of cheesecloth, filtered though a 0.45 μM filter, dialyzed overnight at 4 °C against HMfB storage buffer (50 mM Tris-HCl, pH 8.0, 333 mM sodium citrate) and stored at 4 °C. This ‘crude extract’ was used in the ligations without further purification.

### Generation and purification of minicircle topoisomers

Plasmid pMC336, constructed by deleting three base pairs from pMC339-BbvCI (ref. 14) using the QuikChange II site-directed mutagenesis kit (Stratagene, La Jolla, CA), generates both the 336 bp and 672 bp minicircles. Plasmid sequences were confirmed by DNA sequencing (Lonestar Labs, Houston, TX). DNA minicircles (minivectors™) were purchased from Twister Biotech (Houston, TX). Minicircle DNA was nicked at a single site using nicking endonuclease Nb.BbvCI according to the manufacturer’s protocol. The reaction was subsequently incubated at 80 °C to inactivate the nicking endonuclease, extracted with 25:24:1 phenol:chloroform:isoamyl alcohol, extracted with chloroform, precipitated with ethanol, and resuspended in TE buffer (10 mM Tris-HCl, pH 8.0, and 1 mM disodium Ethylenediaminetetraacetic acid (EDTA)). Negatively supercoiled topoisomers were generated using ethidium bromide as described^14^. Positively supercoiled topoisomers were prepared using HMfB crude extract. The method of generating supercoiling did not influence the structure of the minicircles (Supplementary Fig. 1b). Optimal HMfB:DNA ratios were empirically determined for each HMfB preparation. Nicked minicircle DNA was incubated in the presence of HMfB crude extract for 15 min in 50 mM Tris-HCl, pH 7.5, 10 mM MgCl_2_, 1 mM ATP and 10 mM DTT. T4 DNA ligase was added and the reaction mix was incubated for 4 h at 32 °C. Disodium EDTA (20 mM) was added to quench ligation, followed by the addition of RNase A (50 μg ml^−1^ ) and the mixture was incubated at 37 °C for 30 min. Proteinase K (50 μg ml^−1^ ) was added and the reaction was incubated at 45 °C for 1 h in the presence of 1% SDS. The mixture was concentrated and desalted using an Amicon centrifugal filter (EMD Millipore, Billerica, MA), precipitated with ethanol, and the DNA was resuspended in TE buffer (pH 8.0).

Individual minicircle topoisomers were separated by electrophoresis in 5% polyacrylamide gels (acrylamide:bis-acrylamide=29:1) at 125 V (∼6 V cm^−1^ ) for 8 h in the presence of Tris-acetate buffer (pH 8.2) containing 10 mM CaCl_2_ (for negatively supercoiled topoisomers), 1 mM disodium EDTA (for positively supercoiled topoisomers) or 1 mM disodium EDTA with 2 μg ml^−1^ ethidium bromide (for the relaxed, *Lk*=32, topoisomer). Preparative gels were stained with ethidium bromide. DNA was electroeluted from the gel slices at 80 V for ∼16 h at room temperature in D-tube dialyzers (Novagen, Madison, WI) in 40 mM Tris-acetate buffer (pH 8.2) with 1 mM disodium EDTA. Electroeluted DNA was extracted thrice with 1-butanol to remove residual ethidium bromide, extracted with chloroform, precipitated with ethanol, resuspended in 10 mM Tris-HCl, pH 8.0, and 0.1 mM disodium EDTA, desalted using an Amicon 0.5 ml centrifugal filter, precipitated again in ethanol, and resuspended in 10 mM Tris-HCl, pH 8.0, and 0.1 mM disodium EDTA. DNA concentrations were determined using a Nanodrop spectrophotometer (Thermo Scientific, Wilmington, DE). DNA samples were analysed by electrophoresis through 5% polyacrylamide gels (acrylamide:bis-acrylamide=29:1) in Tris-acetate buffer (pH 8.2) containing 10 mM CaCl_2_ at 125 V (∼6 V cm^−1^ ) for ∼8 h. Buffer was recirculated continuously throughout electrophoresis. Gels were stained with SYBR Gold (Life Technologies, Grand Island, NY) and visualized using a FOTO/ANALYST Investigator imaging system (Fotodyne, Hartland, WI) with quantification using the TotalLab software (TotalLab, Newcastle, UK).

### Relaxation assay with hTopoIIα

Minicircle topoisomers (336 bp, 25 ng) were incubated with hTopoIIα (10:1 DNA:enzyme molar ratio) in 10 mM Tris-HCl, pH 7.9, 175 mM KCl, 0.1 mM disodium EDTA, 5 mM MgCl_2_ and 2.5% glycerol in a total reaction volume=20 μl. ATP was added to a final concentration of 1 mM to start relaxation, and the reaction mixes were incubated at 37 °C for 20 min. Reactions were stopped by the addition of 3 μl of 50 mM disodium EDTA and 5% SDS (final concentration, 6.5 mM and 0.7%, respectively). Proteinase K was added (final concentration=1 μg μl^−1^ ) and the mixtures were incubated for 30 min at 45 °C. DNA topoisomers were resolved on a 5% polyacrylamide gel (acrylamide:bis-acrylamide=29:1) in 40 mM Tris-acetate containing 10 mM CaCl_2_ for 60 V for ∼16.5 h. Buffer was recirculated continuously throughout electrophoresis. Gels were destained in 40 mM Tris-acetate containing 10 mM disodium EDTA, followed by subsequent staining with SYBR Gold and visualized using a FOTODYNE FOTO/analyst investigator imaging system.

### Electron cryo-tomography

DNA samples (100 ng μl^−1^) in Tris-acetate buffer (pH 8.2) supplemented with 10 mM CaCl_2_ were applied onto glow-discharged 200-mesh copper Quantifoil R 1.2/1.3 holey carbon grids (Quantifoil Micro Tools GmBH, Großlöbichau, Germany) that had been pretreated with 15 nm gold-BSA fiducials. The grids were blotted and vitrified using a Mark IV Vitrobot (FEI, Hillsboro, OR), and stored in liquid nitrogen. Tilt series were collected on a JEM2200FS 200 kV electron microscope (JEOL, Tokyo, Japan) equipped with a field emission gun, energy filter (set to 20 eV) and a 4 k by 4 k CCD camera (Gatan, Pleasanton, CA). The specimen was maintained at −175 °C in a Gatan Model 626 cryo-holder. The microscope settings were spot size=5, condenser aperture=100 μm and objective aperture=60 μm. Images were collected at ∼25,000x magnification, typically from −60° to +60° nominal tilt, in 3° (or in one case 10°) increments, with intended defocus of 5−7 μm using SerialEM^44^. Final sampling was 4.52 Å pixel^−1^. Tomograms were reconstructed in IMOD^45^ and visualized in 3dmod. Individual DNA minicircle topoisomers were identified by eye in 3dmod and computationally extracted. A Gaussian low-pass filter was applied to each subvolume before visualization in UCSF Chimera^46^.

Within each population, minicircles were classified visually into categories by at least two individuals. Differences among categories were sometimes subtle. The difference between the racquet and the needle, for example, was only in the relative sizes of the loop and the handle. Variations within the needle populations were in the size of the loop and amount of bending of the minicircle. Minicircles were classified as ‘Other’ if they did not conform to the shapes shown in Fig. 1c. Five of the most prominent alternative configurations are shown in Fig. 1d; however, we also observed densities that appeared to be minicircles, but their shapes were difficult to decipher.

### Computational tracing of DNA minicircle

First, cryo-ET density maps were normalized, low-pass filtered, and disconnected noise was removed computationally^47^. Initially, five connected vertices in a closed loop were placed around each minicircle. A score was calculated including the density value at the vertices, the length of each edge, and the angle between the edges. Each vertex was then iteratively moved in the direction of the density gradient to optimize the score, with the length and angle terms acting as restoring forces to cause the shape to remain as much like an open loop as permitted by the density map. After this initial optimization, the number of vertices was doubled by adding a point in the middle of each edge. The set of vertices was subsequently optimized in the same way and the procedure was repeated once more with 20 vertices. The convergence of the algorithm was verified using multiple random starting locations and assessing the similarity of results. Relative axis lengths were computed from the coordinates of the final polygon for each minicircle.

To compute the radius of gyration, the raw density for each minicircle was extracted by directly including any voxels within 45 Å of the fit path, then damped to a Gaussian decay extending to a distance of 180 Å. The radius of gyration was then calculated from the extracted subvolume using the position and density of each voxel, making no direct use of the fit vertices.

### Molecular dynamics simulations

Starting structures of 336-bp minicircle DNA with different helical twists (corresponding to seven *Lk* values of 27–33) were built using the NAB module implemented in AMBERTOOLS11 (ref. 48). The AMBER99 force field with parmbsc0 corrections for the α and γ dihedral parameters^49^ and a correction for χ (glycosidic bond) parameters^50^ was used to describe the DNA. Following a multistage equilibration protocol previously described^51^, these starting structures were subjected to 20 ns of implicitly solvated MD using the Tsui and Case Generalized Born/Solvent Accessible area (GB/SA) method^52^ at 300 K and 100 mM monovalent salt concentration, which is roughly equivalent to 10 mM CaCl_2_ (ref. 53), with the long-range electrostatic cutoff set to 100 Å. Restraints were imposed on the hydrogen bonding interactions between complementary base pairs. To determine the superhelical density of each of the seven topoisomers, we identified the relaxed topoisomer (which must be the least compact minicircle) by calculating the radius of gyration (Fig. 3a). In the simulations, Δ*Lk*=0 actually corresponds to an *Lk* of 30 (reflecting the known underestimation of relaxed DNA twist in the AMBER forcefield^51^).

We performed these initial MD simulations with a continuum representation of the solvent to more rapidly explore conformational space in the absence of any frictional drag from collisions with water molecules. Discarding the first 5 ns for equilibration, the calculated average writhe for each topoisomer did not change by more than 8% for each topoisomer when the simulations were extended from 15 to 20 ns; consequently we considered the writhed to be adequately sampled by the implicitly solvated simulations (Supplementary Fig. 4). We then performed clustering analysis using the average linkage algorithm^54^ in the PTRAJ package in AMBERTOOLS11. A representative structure of the most populated cluster was chosen and solvated in 100 mM Na^+^ and Cl^-^ counterions^55^ and a TIP3P water box^56^, and 10 ns MD simulation runs were performed using the GROMACS 4.5 program^57^ with standard MD protocols (Supplementary Information). By comparing the maximum relative speeds of the centres of mass of two DNA bases located on opposite sides of the minicircle in implicit and explicitly solvated simulations, we estimated that inclusion of the frictional term from solvent/solute interactions retards the dynamics of the DNA by about an order of magnitude. This difference suggests that the timescale required for interconversion among minicircle conformers lies between 100 ns and a microsecond. We then calculated RMSDs between the simulated atomistic models in explicit solvent and the ensemble of computational traces obtained from cryo-ET, and selected the four MD models with the lowest RMSD for direct comparison with the cryo-ET density maps (Fig. 3 and Supplementary Movie 10–13).

### Nuclease Bal-31 assay

Minicircle DNA (300 ng in 60 μl final volume) was incubated with 0.3 units of nuclease Bal-31 at 30 °C in 20 mM Tris-HCl, pH 8.0, 600 mM NaCl, 12 mM MgCl_2_, 12 mM CaCl_2_ and 1 mM disodium EDTA. At 1, 10, 20 and 60-minute intervals, 10 μl (50 ng) samples were removed, quenched by addition of an equal volume of stop buffer (50 mM Tris-HCl, pH 8.0, 100 mM disodium EDTA, 10% glycerol, 200 μg ml^−1^ proteinase K), followed by incubation at 45 °C for 30 min to degrade Bal-31. Products were analysed by electrophoresis through 5% polyacrylamide gels (acrylamide:bis-acrylamide=29:1) in Tris-acetate buffer (pH 8.2) containing 1 mM disodium EDTA. Gels were run for 4 h at 125 V (∼6 V cm^−1^). Buffer was recirculated continuously throughout electrophoresis. Gels were stained with SYBR Gold and visualized using a FOTO/ANALYST Investigator imaging system with quantification using the TotalLab software.

### Mapping Bal-31 cleavage

Minicircle DNA (7.5 μg of the Δ*Lk*=−6 topoisomer in 3 ml final volume) was incubated with 7.5 units of nuclease Bal-31 at 30 °C in 20 mM Tris-HCl, pH 8.0, 600 mM NaCl, 12 mM MgCl_2_, 12 mM CaCl_2_ and 1 mM disodium EDTA. After 1 min the reaction was quenched by the addition of disodium EDTA (50 mM final concentration) and incubated with proteinase K (100 μg ml^−1^ ) at 45 °C for 1 h. The reaction was concentrated and desalted using an Amicon centrifugal filter. Full length linearized DNA was isolated on 5% polyacrylamide gels (acrylamide:bis-acrylamide=29:1) at 125 V (∼6 V ) for 4 h in the presence of Tris-acetate buffer (pH 8.2) containing 1 mM disodium EDTA. The preparative gel was stained with ethidium bromide and the DNA recovered from the gel as described above.

Bal-31 linearized DNA (100 ng) was incubated with EcoRV, BbvCI or NdeI according to the manufacturer’s protocol. Control reactions were performed with minicircle DNA supplied by Twister Biotech, Inc. (an approximately equal mixture of Δ*Lk*=−2 and Δ*Lk*=−3 topoisomers). Products were analysed by electrophoresis on a 3% agarose gel (NuSieve 3.1 agarose, Lonza, Basel, Switzerland) at 100 V cm^−1^ for 2.5 h in Tris-acetate buffer (pH 8.2) containing 1 mM disodium EDTA. The gel was stained with SYBR Gold and visualized using a FOTO/ANALYST Investigator imaging system. Fragment sizes were determined by measuring distances migrated (measured from the well to the centre of each band using the TotalLab) and compared to a standard curve generated from the bands in the low molecular weight DNA ladder.

### Glyoxal assay

Glyoxal was first deionized with AG-501-X8 mixed bed ion-exchange resin (Bio-Rad, Hercules, CA). 50 ng of minicircle DNA was incubated with 1 M glyoxal in 10 mM sodium phosphate, pH 7.0, for 16 h at room temperature. Control reactions were incubated in 10 mM sodium phosphate only. Samples were analysed by electrophoresis through 5% acrylamide gels (acrylamide:bis-acrylamide=29:1) in 10 mM sodium phosphate buffer (pH 7.0). Gels were run at 75 V for 6 h. Buffer was recirculated continuously throughout electrophoresis. Gels were stained with SYBR Gold and visualized using a FOTO/ANALYST Investigator imaging system. The diffuse spread of the negatively supercoiled topoisomers incubated with glyoxal precluded accurate quantitation of the data.

## Additional information

**Accession codes:** Cryo-ET maps of representative minicircles of various shapes have been deposited into the EMDB under accession code EMD-6462.

**How to cite this article:** Irobalieva, R. N. *et al*. Structural diversity of supercoiled DNA. *Nat. Commun.* 6:8440 doi: 10.1038/ncomms9440 (2015).

## Supplementary Material

Supplementary Information Supplementary Figures 1-5, Supplementary Tables 1-3, Supplementary Note 1 and Supplementary References

Supplementary Movie 1 An example of a tomogram obtained by cryo-ET. Dark densities represent volume rendering of the DNA minicircles. Red overlay was achieved by segmentation of the molecules.

Supplementary Movie 2 Structure of an individual minicircle obtained by cryo-ET. The example shown adopts the handcuffs conformation.

Supplementary Movie 3 Structure of an individual minicircle obtained by cryo-ET. The example shown adopts the needle conformation.

Supplementary Movie 4 Implicit molecular dynamics simulation of ?Lk = -2.

Supplementary Movie 5 Implicit molecular dynamics simulation of ?Lk = +1.

Supplementary Movie 6 Explicit molecular dynamics simulation showing an open circle conformation.

Supplementary Movie 7 Explicit molecular dynamics simulation showing a figure-8 conformation.

Supplementary Movie 8 Explicit molecular dynamics simulations showing a racquet conformation.

Supplementary Movie 9 Explicit molecular dynamics simulations showing a handcuffs conformation.

Supplementary Movie 10 Atomistic MD structures from the explicitly solvated MD simulations embedded within the cryo-ET density maps showing the open circle conformation (Movie 10: RMSD = 26.4 Å, 8%). (The percentage value refers to the RMSD compared to the longest dimension of the tomogram).

Supplementary Movie 11 Atomistic MD structures from the explicitly solvated MD simulations embedded within the cryo-ET density maps showing the figure-8 conformation (Movie 11: RMSD = 39.4 Å, 11%). (The percentage value refers to the RMSD compared to the longest dimension of the tomogram)

Supplementary Movie 12 Atomistic MD structures from the explicitly solvated MD simulations embedded within the cryo-ET density maps showing the racquet conformation (Movie 12: RMSD = 35.5 Å, 10%). (The percentage value refers to the RMSD compared to the longest dimension of the tomogram)

Supplementary Movie 13 Atomistic MD structures from the explicitly solvated MD simulations embedded within the cryo-ET density maps showing the handcuffs conformation (Movie 13: RMSD = 28.3 Å, 7%). (The percentage value refers to the RMSD compared to the longest dimension of the tomogram).
