# Erratum: Structural diversity of supercoiled DNA

**DOI:** 10.1038/ncomms9851

**Published:** 2015-10-29

**Authors:** Rossitza N. Irobalieva, Jonathan M. Fogg, Daniel J. Catanese, Thana Sutthibutpong, Muyuan Chen, Anna K. Barker, Steven J. Ludtke, Sarah A. Harris, Michael F. Schmid, Wah Chiu, Lynn Zechiedrich

10.1038/ncomms9440

The original version of this Article contained an error in the spelling of the author Daniel J. Catanese Jr, which was incorrectly given as Daniel J. Catanese. This has now been corrected in both the PDF and HTML versions of the Article.

