# Supplementary material for: Structural diversity of supercoiled DNA
Source: Nat Commun. 2015 Oct 12;6:8440. doi: 10.1038/ncomms9440 (PMC4608029; doi:10.1038/ncomms9440)
Supplement: Supplementary Information — Supplementary Figures 1-5, Supplementary Tables 1-3, Supplementary Note 1 and Supplementary References [file ncomms9440-s1.pdf]

Supplementary Figures

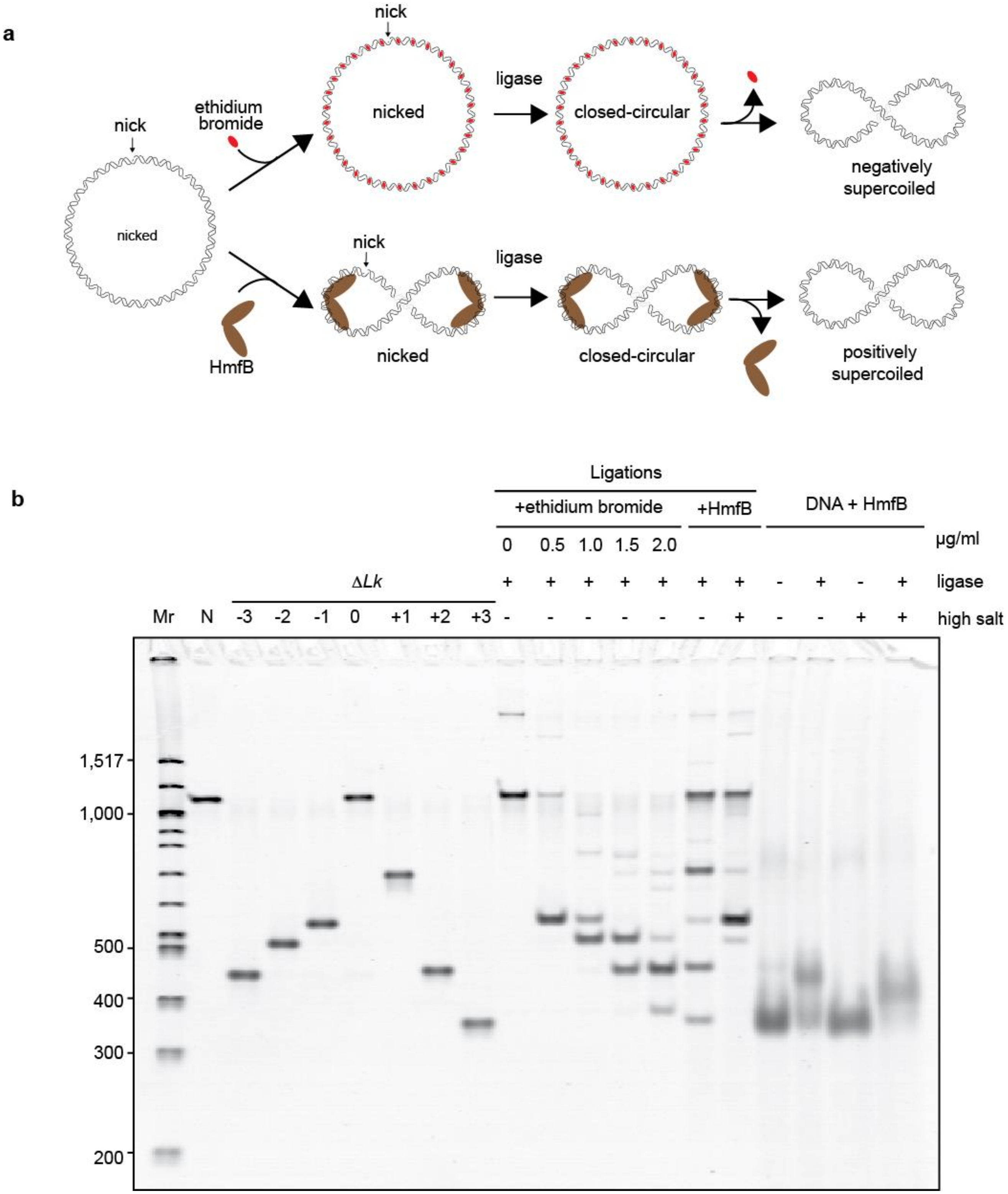

Supplementary Figure 1 | Minicircle topoisomer generation. a, Generation of supercoiled topoisomers. Minicircles were nicked with the sequence-specific nicking endonuclease, Nb.BbvCI.

Addition of ethidium bromide unwinds the DNA. Minicircle DNA is wrapped by HMfB, stabilizing positive writhe. Ligase seals the nicks and traps the induced supercoiling. Removal of ethidium bromide or HMfB allows the supercoiling to repartition between twist and writhe. **b,** Differences between negatively and positively supercoiled minicircle DNA do not result from the differences in their generation and purification. Negatively supercoiled topoisomers are generated using ethidium bromide and cleaned up using chemical extraction prior to gel electrophoresis. Positive supercoiling is generated by HMfB under low salt conditions. Negative supercoiling by HMfB occurs under high salt conditions. Nicked minicircles were religated in T4 DNA ligase buffer in the presence of HMfB in ligase buffer alone (low salt) or ligase buffer supplemented with 350 mM sodium glutamate (high salt), and subsequently cleaned up using proteinase K. The electrophoretic mobility of the negatively supercoiled topoisomers generated using HMfB was identical to that of topoisomers generated using ethidium bromide. The right four lanes do not include the proteinase K digestion. Markers: Mr: 100 bp ladder, N: nicked 336 bp minicircle.

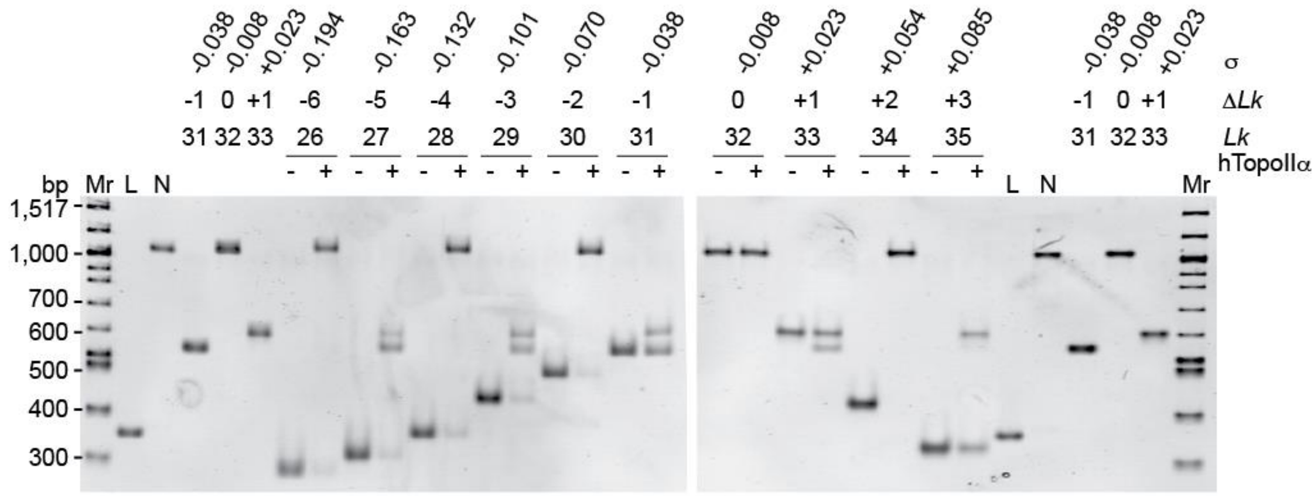

Supplementary Figure 2 | Human topoisomerase IIα (htopoIIα) relaxation of 336 bp DNA minicircles as a function of supercoiling. Minicircle topoisomers were relaxed with htopoIIα as indicated (+). Reaction products were separated on a 5% polyacrylamide gel in 40 mM Tris-acetate buffer containing 10 mM $CaCl_2$. Mr: 100 bp ladder, L: 336 bp linear DNA, N: nicked 336 bp minicircle. These same results were obtained twice.

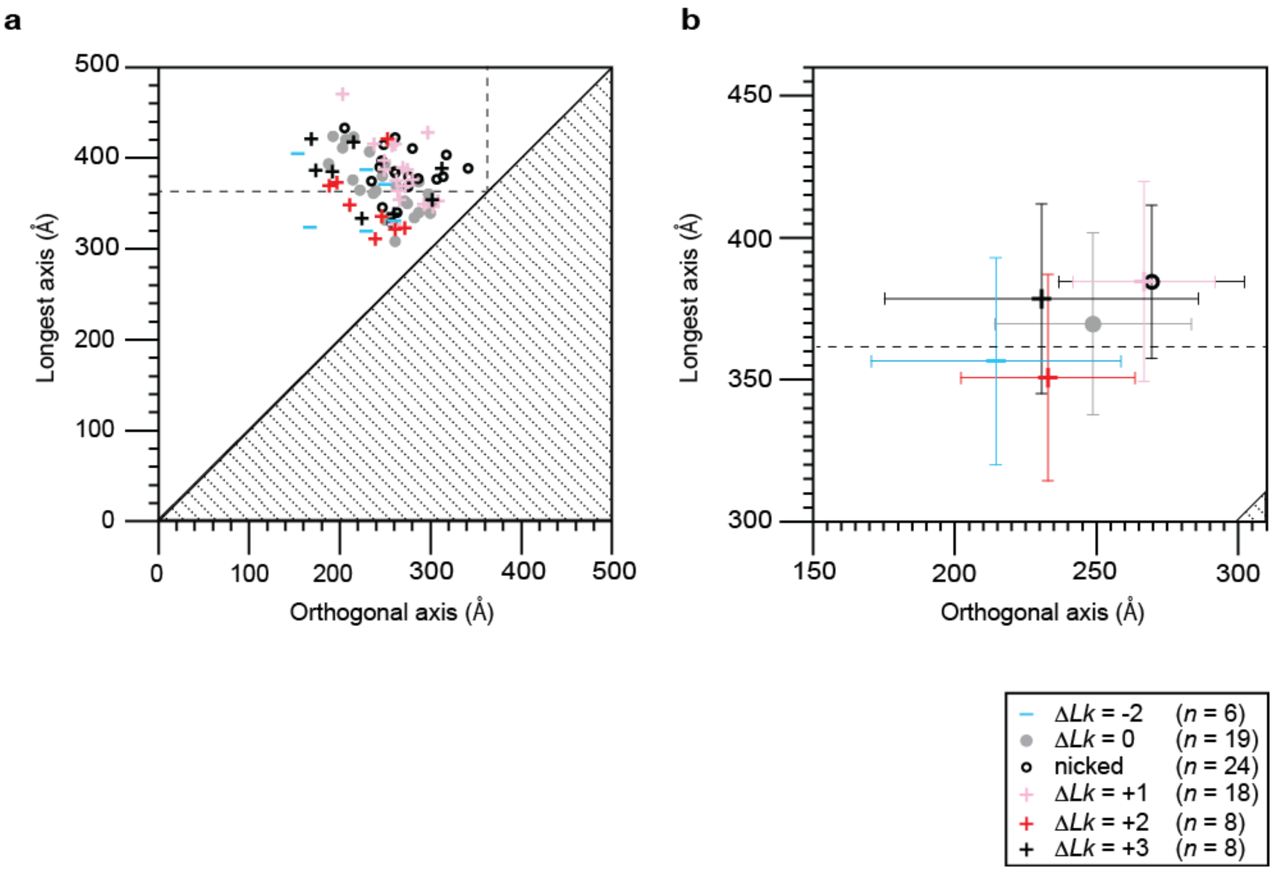

Supplementary Figure 3 | Dimensions of open circle minicircles as a function of supercoiling. From a random, unbiased selection of 336 bp planar open circles, the longest axis and the axis orthogonal to the longest axis were measured computationally and by three people independently. **a**, These four values were averaged for each open circle and the longest axis values were plotted against the values for the orthogonal axis. The dashed lines show the theoretical diameter of a perfectly circular 336 bp minicircle, assuming a rise per base pair of 3.4 Å. **b**, Average dimensions of the open circles for each topoisomer. Error bars indicate one standard deviation. The dashed line is the same as in **a**.

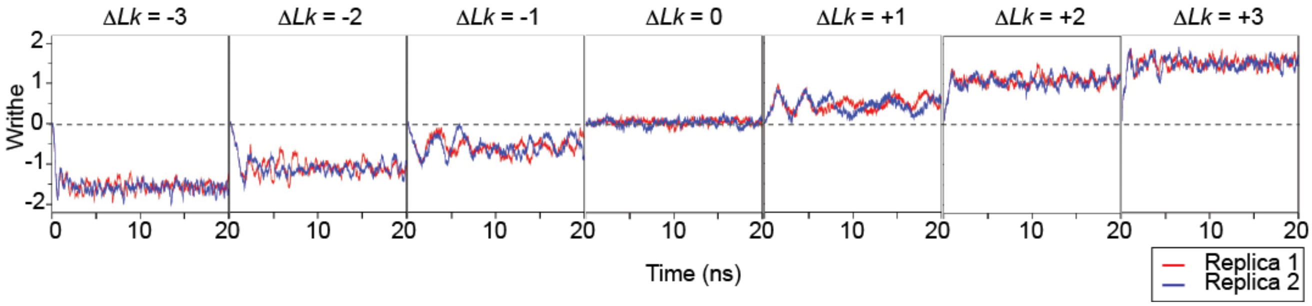

Supplementary Figure 4 | DNA writhe during MD simulations in implicit solvent. The writhe of the DNA minicircles was calculated throughout the trajectory for each of the seven topoisomers (in red). In order to demonstrate that the results are reproducible, we performed replica simulations (in blue) using identical simulation conditions but different starting velocities sampled from a Maxwell-Boltzmann distribution at 300 K. Comparing the red and the blue curves shows that the initial and replica simulations sample equivalently writhed conformations for each individual topoisomer.

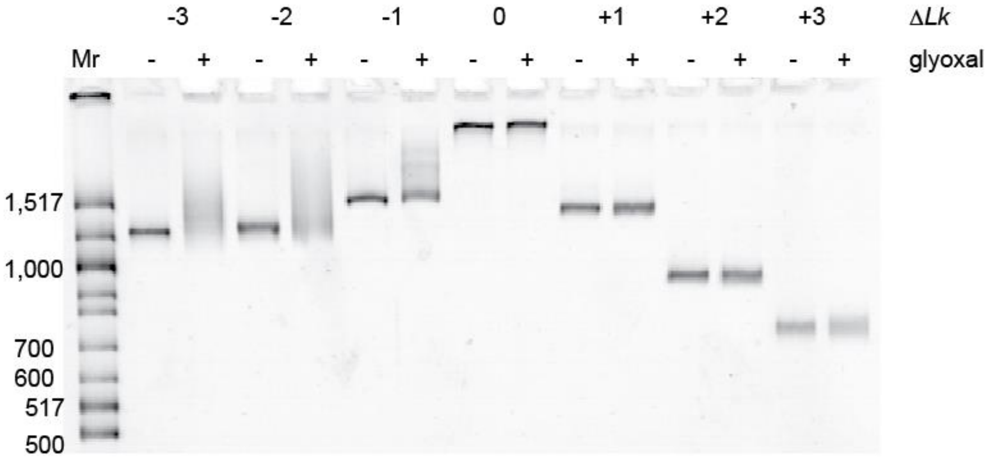

Supplementary Figure 5| Trapping of localized denaturation with glyoxal. The topoisomers indicated were incubated overnight in the absence (-) or presence (+) of glyoxal and analyzed by polyacrylamide gel electrophoresis. Glyoxal binds to and stabilizes exposed bases in DNA, preventing renaturation, resulting in reduced electrophoretic mobility. Mr: 100 bp DNA ladder.

Supplementary Tables

Minicircle Designation	***Lk***	**Δ*Lk***	***σ***
Nicked	~32.25	0	0
Δ*Lk* = -6	26	-6.25	-0.194
Δ*Lk* = -5	27	-5.25	-0.163
Δ*Lk* = -4	28	-4.25	-0.132
Δ*Lk* = -3	29	-3.25	-0.101
Δ*Lk* = -2	30	-2.25	-0.070
Δ*Lk* = -1	31	-1.25	-0.039
Δ*Lk* = 0	32	-0.25	-0.008
Δ*Lk* = +1	33	+0.75	+0.023
Δ*Lk* = +2	34	+1.75	+0.054
Δ*Lk* = +3	35	+2.75	+0.085

Supplementary Table 1| Values for Δ*Lk* and σ

Values were calculated as described in Supplementary Note 1.

Equilibration stage	All-atom DNA restraints	Time
Minimization 1	k = 500.0 kCal/mol/Å^2	10,000 cycles
Minimization 2	No restraints	10,000 cycles
Equilibration 1	k = 500.0 kCal/mol/Å^2, T = 100K	10 ps
Equilibration 2	k = 50.0 kCal/mol/Å^2, T = 300K	10 ps
Equilibration 3	k = 25.0 kCal/mol/Å^2, T = 300K	10 ps

Supplementary Table 2 | Force constants of the coordinate restraints and durations of the minimization and equilibration steps in implicit solvents MD simulations.

After the last equilibration stage, the structure was subjected to the production run with all the Watson-Crick hydrogen bonds restrained.

Equilibration stage	All-atom DNA restraints	Time
Minimization 1	k = 500.0 kCal/mol/$Å^2$	10,000 cycles
Minimization 2	k = 50.0 kCal/mol/$Å^2$	10,000 cycles
Minimization 3	k = 25.0 kCal/mol/$Å^2$	10,000 cycles
Minimization 4	No restraints	10,000 cycles
Equilibration 1	k = 500.0 kCal/mol/$Å^2$, T = 100K	10 ps
Equilibration 2	k = 50.0 kCal/mol/$Å^2$, T = 300K	10 ps
Equilibration 3	k = 50.0 kCal/mol/$Å^2$, T = 300K	10 ps
Equilibration 4	k = 25.0 kCal/mol/$Å^2$, T = 300K	10 ps
Equilibration 5	k = 10.0 kCal/mol/$Å^2$, T = 300K	10 ps
Equilibration 6	k = 5.0 kCal/mol/$Å^2$, T = 300K	10 ps
Equilibration 7	k = 2.5 kCal/mol/$Å^2$, T = 300K	10 ps
Equilibration 8	k = 1.0 kCal/mol/$Å^2$, T = 300K	10 ps

Supplementary Table 3| Force constants of the coordinate restraints and durations of the minimization and equilibration steps in explicit solvent MD simulations. After the last equilibration stage, the structure was subjected to the production run with no restraints.

Supplementary Note 1

Calculation of Lk, ΔLk and σ: Although Lk_0, and therefore ΔLk, are not necessarily integral for simplicity we refer to each topoisomer in the main text by its ΔLk value rounded to the nearest integer. More precise values for these parameters can be found above (Supplementary Table 1). The helical repeat of DNA depends on the solution conditions. Divalent metal ions overwind the DNA helix significantly[1] and consequently the DNA sequence used in the study has a helical repeat ~10.42 bp/turn in 10 mM $CaCl_2$ (Fogg *et al.*, manuscript in preparation). Therefore, the linking number of a hypothetically relaxed DNA molecule; $Lk_0 = 336/10.42 = 32.25$. In the absence of ethidium bromide or HmfB, the major topoisomer generated is $Lk = 32$, the topoisomer closest to Lk_0. Negatively supercoiled topoisomers are generated by varying the concentration of ethidium bromide to change Lk in steps of 1 as described previously[2] and as shown (Extended Data Fig. 1b). For positively supercoiled topoisomers, a mixture of topoisomers are generated (Extended Data Fig.1b). Each major band corresponds to a distinct topoisomer (Extended Data Fig.1b). For each topoisomer the linking difference (ΔLk) is calculated relative to Lk_0. The linking difference (ΔLk) of each topoisomer was determined relative to Lk_0 ($\Delta Lk = Lk - Lk_0$). The ΔLk is scaled to the size of the DNA to give the superhelical density (σ) = $\Delta Lk/Lk_0$.

Supplementary References

1. Xu, Y. C. & Bremer, H. Winding of the DNA helix by divalent metal ions. *Nucleic Acids Res.* **25,** 4067–4071 (1997).

2. Fogg, J. M. *et al.* Exploring writhe in supercoiled minicircle DNA. *J. Phys. Condens. Matter Inst. Phys. J.* **18,** S145–S159 (2006).
